# Mobile augmented reality based indoor map for improving geo-visualization

**DOI:** 10.7717/peerj-cs.704

**Published:** 2021-09-02

**Authors:** Wei Ma, Shuai Zhang, Jincai Huang

**Affiliations:** 1 School of Civil Engineering, Chongqing Jiaotong University, Chongqing, Nanan, China; 2 Big data Institute, Central South University, Changsha, Yuelu, China

**Keywords:** Mobile augmented reality, BLE and PDR fusion, Indoor map, Indoor localization, Geo-visualization

## Abstract

Unlike traditional visualization methods, augmented reality (AR) inserts virtual objects and information directly into digital representations of the real world, which makes these objects and data more easily understood and interactive. The integration of AR and GIS is a promising way to display spatial information in context. However, most existing AR-GIS applications only provide local spatial information in a fixed location, which is exposed to a set of problems, limited legibility, information clutter and the incomplete spatial relationships. In addition, the indoor space structure is complex and GPS is unavailable, so that indoor AR systems are further impeded by the limited capacity of these systems to detect and display location and semantic information. To address this problem, the localization technique for tracking the camera positions was fused by Bluetooth low energy (BLE) and pedestrian dead reckoning (PDR). The multi-sensor fusion-based algorithm employs a particle filter. Based on the direction and position of the phone, the spatial information is automatically registered onto a live camera view. The proposed algorithm extracts and matches a bounding box of the indoor map to a real world scene. Finally, the indoor map and semantic information were rendered into the real world, based on the real-time computed spatial relationship between the indoor map and live camera view. Experimental results demonstrate that the average positioning error of our approach is 1.47 m, and 80% of proposed method error is within approximately 1.8 m. The positioning result can effectively support that AR and indoor map fusion technique links rich indoor spatial information to real world scenes. The method is not only suitable for traditional tasks related to indoor navigation, but it is also promising method for crowdsourcing data collection and indoor map reconstruction.

## Introduction

### Research background

Consumer applications, like smart phones integrated with GIS, could provide users with useful information. This geospatial information however, might be difficult to understand. Data pertaining to complex indoor environments is especially challenging for users with limited geographic knowledge, but visualization enhances situational awareness among users for a more satisfying experience. Thus, in either 2D to 3D representation, flexibility and realism requirements for spatial information visualization are becoming ever more demanding. In contrast to traditional visualization methods, AR combines virtual objects with a real scene, as an intuitive way to convey information that is otherwise difficult to transmit (Carrera et al., 2017). The integration of AR and GIS promotes a more expressive way to display GIS data in context, closely related to information about the surrounding environment.

Researchers have made some useful explorations in AR-GIS (Huang, Sun & Li, 2016; Wang et al., 2014). Ruta et al. (2016) proposed an AR system for description of Location-Based Services (LBSs) and Points of Interest (POIs), which supports users with disabilities. Liu, Jonsson & Seipel (2020) presented an AR system for infrared thermographic façade inspection. The system employed a third person perspective augmented view displayed and camera tracking based on image registration. Sun et al. offered an interactive method based on the linear characteristics to combine 3D GIS with outdoor AR (Shaughnessy & Resnick, 2013). Data management between GIS databases and AR visualizations maintains an interactive data round-trip (Zollmann et al., 2012). Liu et al. integrated AR and Location-based social networks for enriching the GIS-science research agenda in data conflation and multimedia storytelling (Chengbi & Sven, 2018). However, there are still several open challenges for spatial information AR visualization, especially in indoor space where the structure layout complex and mobile phone sensors limited.

AR and GIS techniques have received increasing attention. Thus there is a need to better understand the particulars of AR interfaces in indoor environments beyond location-awareness affecting GIS data visualization and interaction performance in AR-GIS applications. Camera pose tracking is the keystone for accurate understanding of spatial relationships in AR visualization (Sadeghi-Niaraki & Choi, 2020). To overcome the limitations of GPS indoors, many researchers have focused on the vision-based tracking methods, such as Structure from Motion (SFM) or Simultaneous Localization and Mapping (SLAM) (Liu, Zhang & Bao, 2016). These camera pose tracking methods rely heavily on the reference points. Xu et al. (2020) proposed a Walkability network-based Augmented Reality (WaNAR). But the method needs a man-made 3D drawing of indoor walkable space. Indoor environments often contain scenes without visual features such as bare walls or windows, thus the indoor accuracy is seriously limited. Thus, a light weight and accurate indoor camera pose tracking method suitable for AR is still an elusive goal.

The main challenge is that most existing AR applications are usually used for short periods of time and for specific purposes, they do not allow for a continuous, universal, context-aware AR experience, except that there is still a lack of effective organization in the dynamic spatial information visualization in AR-GIS system. It leads to spatial information clutter and limited visibility due to fixed location. Visualizing spatial data in an indoor AR environment requires special consideration because a disproportionate mixture of AR content or redundant information will mislead and confuse users. Therefore, organizing these dynamic spatial information and visual elements is another challenge.

### Aim of the study

In this paper, the objective is optimizing and enriching the spatial information visualization in indoor environments. We bridge the indoor map and situational visualization with real world scene based on mobile augmented reality technique. Firstly, we divide an indoor map by a regular grid and build the index of map data to diminish redundant data and limit AR visualization within a certain range of the current location. At the camera tracking stage, we propose a novel multi-sensor fusion-based algorithm, which employs a particle filter to fuse BLE and PDR. Based on the direction and position of the phone, the extent of an indoor map visible in the camera view is extracted and the spatial entities calculated just for this area. Considering different poses of the mobile phone in users’ hand, we also designed changed forms of the AR view. After applying a coordinate transformation to the spatial entities, the fused AR-GIS system renders spatial information in relation to the area visible in the dynamic camera view.

### Contributions

The contributions can be summarized as follows:

• We propose a novel AR-GIS method for indoor environments that fuses an indoor map with dynamic situated visualizations.
• We designed an online indoor positioning method that fuses the BLE and PDR for AR camera tracking.
• We designed a flexible AR system that accessibly visualizes a variety of types of spatial information regardless of the pose of a mobile phone.

### Organization of the paper

The organization of this paper is as follows. The related works are briefly reviewed in Section 2. Section 3 discussed the main methods. The experimental results and analysis are described in Section 4. The conclusions are then presented in Section 5. The Abbreviation table is shown in Table 1.

**Table 1:** Abbreviation table.

Abbreviation	Whole words
AR	Augmented Reality
BLE	Bluetooth low energy
PDR	Pedestrian dead reckoning
GIS	Geographic Information System
LBSs	Location-Based Services
3D GIS	3-Dimensional Geographical Information System
SFM	Structure from Motion
SLAM	Simultaneous Localization and Mapping
SIFT	Scale-Invariant Feature Transform
WaNAR	Walkability network-based Augmented Reality
DoF	Degrees of freedom
GPS	Global Positioning System
IMU	Inertial Measurement Unit
WKNN	Weight k-nearest neighbour
RSS	Received signal strength
PF	Particle filter
CDF	Cumulative Distribution Function

## Related Work

AR has gradually emerged as a popular way to display LBSs. Hence, researchers are focused on AR techniques for GIS visualization in two major areas; camera tracking techniques for estimating camera poses and tracking target objects, and AR visualization to render virtual data onto a live camera view.

### Camera tracking techniques

Indoor localization enables AR camera tracking. The method based on visual features from images was representative way for AR. Skrypnyk & Lowe (2004) established 3D model by multi-view correspondences and localized object for AR based on the Scale-Invariant Feature Transform (SIFT) descriptor. However, vision-based method is difficult to remain accurate and robust in an AR camera-tracking environment. These methods tend to be failed in texture-less environments.

In order to solve this problem, Subakti (2016) designed and utilized visible/invisible markers to determine the indoor position. Zhou et al. (2020) extracts the activity landmarks from crowdsourcing data and clusters the activity landmarks into different clusters. But, those localization methods need manual intervention, which is can’t be used in AR. Ahmad et al. (2020) aligned the floor plan and collected fingerprints on reference points based on the Microsoft HoloLens. But these methods either involve cost of installing and managing wireless access points or positioning error accumulated with time. Xu et al. (2020) proposed a Walkability network-based Augmented Reality (WaNAR) method to positioning and navigation. But this method needs a man-made 3D drawing of indoor walkable space before being used.

SLAM is a widely solution for AR tracking. Li et al. (2019) improve indoor camera localization by optimized 3D prior map. They integrated RGBD SLAM with a deep learning routine whose training dataset is sequential video frames with labelled camera poses. Reitmayr, Eade & Drummond (2007) developed an AR application for annotation in unknown environments based on an extended SLAM that tracks and estimates high-level features automatically. MARINS is an indoor navigation system developed using the Apple ARKit SDK and an associated SLAM system (Diao & Shih, 2018). However, this method is computational expensiveness, which is hard to apply to a wide range of scenarios.

In addition, some experts consider using sensors embedded in mobile phones for locating, such as WiFi or accelerometer (Zhou et al., 2015). Instead of single tracking method, multiple sources combination is a promising way to improve camera tracking (Li et al., 2016; Liu et al., 2021). Ma et al. (2020) proposed a multi-sensor fusion-based algorithm to improve the precision of indoor localization. The multi-source data include activity detection, PDR, and 3D vision-based localization. However, this method cannot meet the requirement of positioning at real-time. Neges et al. (2017) combined visual natural markers and an IMU to support AR indoor navigation. They used the IMU data to estimate the camera position and orientation when a natural marker-based method is limited. Arth et al. (2011) localized the six DoF pose of a mobile phone by using GPS data and a panoramic view of the environment. Li et al. (2017) integrated monocular camera and IMU measurements to estimate metric distance and localize the mobile device. Hybrid tracking methods improve mobile AR, but there is still a gap to low-latency, high precision AR camera tracking for AR visualization in indoor environments. The camera tracking techniques are summarized in Table 2.

**Table 2:** Camera tracking techniques.

	**Advantage**	**Disadvantage**
Visual feature-based	low-cost, no need for multiple sensors	tend to be failed in texture-less environments
Landmark-based	low-cost, no need for extra equipment, high precision	manual intervention required
SLAM	automatic, no need for pre-identify the scene	computational expensiveness, multi-sensor required,
Multiple sensors-based	high precision	algorithm complicated, pre-establish device required

### AR visualization

AR visualization is not only about rendering computer graphics, but also can help make virtual 2D/3D data easier to understand as an aid to navigation in the real world. For helping designers in managing the aspect of layout and the representation of reasonable AR view, Javornik et al. (2019) compared the different visualization effect between two types of AR content—image and text and image by using Unity 3D and the Vuforia library. Tönnis, Klein & Klinker (2008) considered the impact of the AR visualization style on user perception and designed three types of directional arrows to improve the AR presentation in large distances. They rendered directional arrows according to different speeds. To manage the amount of information in AR view, Mulloni, Dünser & Schmalstieg (2010) proposed two types of zooming interfaces to reduce the user reading load, the egocentric panoramic 360° view and an exocentric top-down view. Tsai & Huang (2017) investigated the AR content presentation and human interaction problems based on the smart glasses. This research provides some interesting references for AR visualization, but the spatial information visualization contains more content and needs more complex visual element.

To solve the problem of limited information visibility, Zollmann, Poglitsch & Ventura (2017) proposed a set of Situated Visualization techniques (White & Feiner, 2009) for a “street-view” perspective. They implemented the dynamic annotation placement, label alignment and occlusion culling for scene information extracted from a GIS database. To resolve the similar problem, Bell, Feiner & Hllerer (2001) presented a 3D representation algorithm for virtual or mixed environments with virtual objects. However, the structural distortions in representations of space are influenced peoples’ spatial perception. Keil et al. (2020) projected a holographic grid into 3D space to explore whether the structural distortions can be reduced. However, their method was based on the additional holograms equipment. Matsuda et al. (2013) proposed a method for providing a non-3D display based on pseudo motion parallax. Considering the user’s point of view, they superimposed computer graphics (CG) images behind the scene display. Grasset et al. (2013) proposed an image-based label placed method that combined a visual saliency algorithm with edge analysis to find the image regions and geometric constraints. Fenais et al. (2019) developed an AR-GIS system for mapping and capturing underground utilities. However, these AR-GIS applications are used for short periods of time and for specific purposes.

The existing AR-GIS applications only provide local spatial information in fixed location for users. The applications with comprehensive spatial information are rarely reported in the literature. These systems do not allow for a continuous and context-aware AR experience. Moreover, the dynamic spatial information visualization in AR-GIS system is tend to clutter and limited visibility due to fixed location.

In this paper, we focus on how to localize and organize the spatial information into an AR view. The goal is to construct a mobile augmented reality system that visualizes spatial information in indoor environment. The proposed AR-GIS system is fused an indoor map and situational visualization based on a mobile phone, which can provide dynamic and comprehensive spatial information for users.

## Methods

### Overview

**Figure 1:**
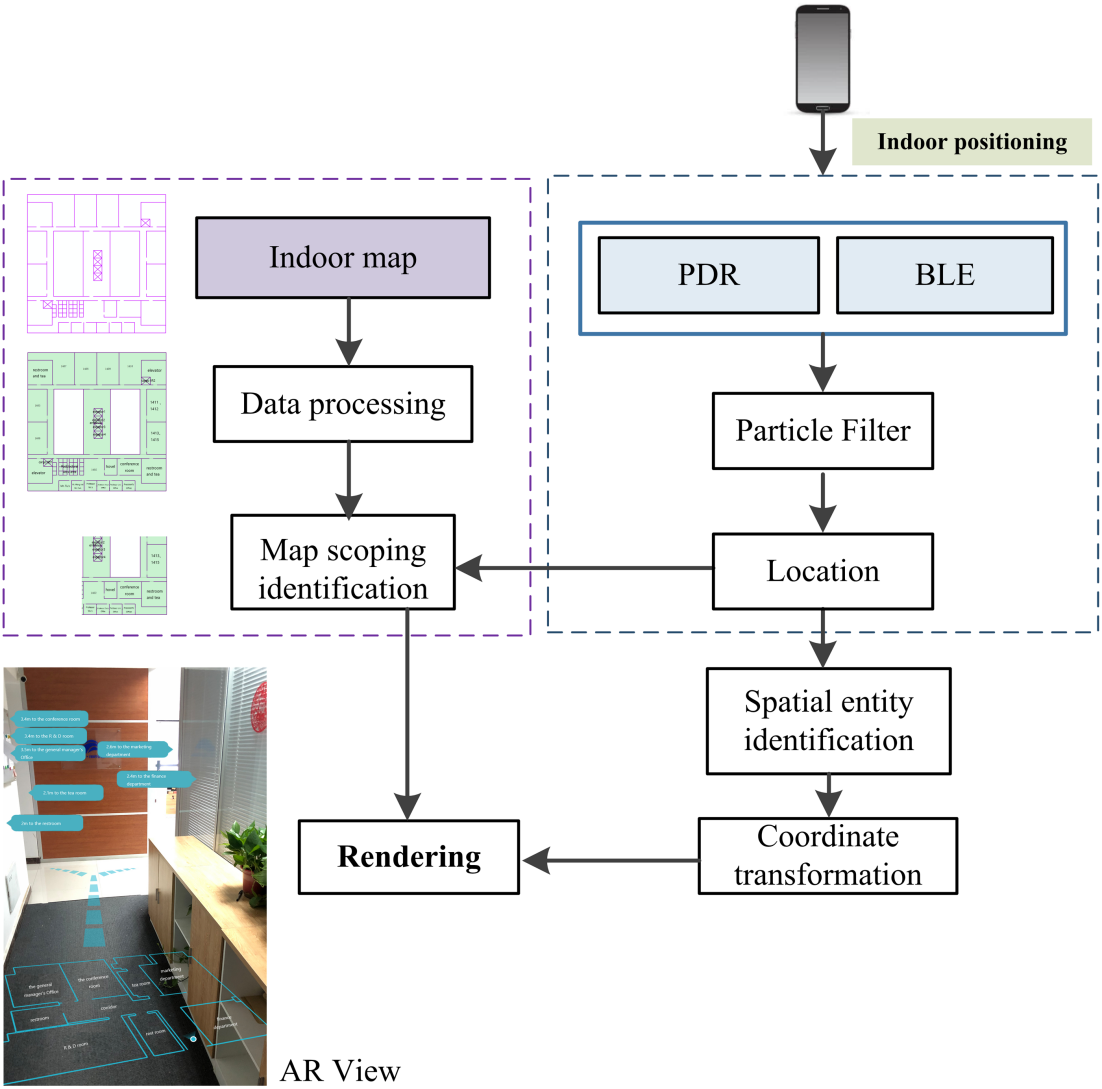
The workflow of augmented reality and indoor map fusion.

The proposed workflow is illustrated in Fig. 1. Given an indoor map from the GIS database, we first divide the indoor map by a regular grid and build an index of map data, to support AR visualization within a certain range at the current user location. At the indoor positioning stage, our novel multi-sensor fusion-based algorithm employs a particle filter to fuse BLE and PDR. We extract the extent of the current location from the indoor map and calculate the spatial entities in front of the camera view according to the direction and position of the phone. The algorithm is proposed to determine screen coordinates for semantic information. The fused mobile augmented reality system renders the semantic information for parts of the indoor model corresponding to the AR camera view.

### Indoor positioning

**Figure 2:**
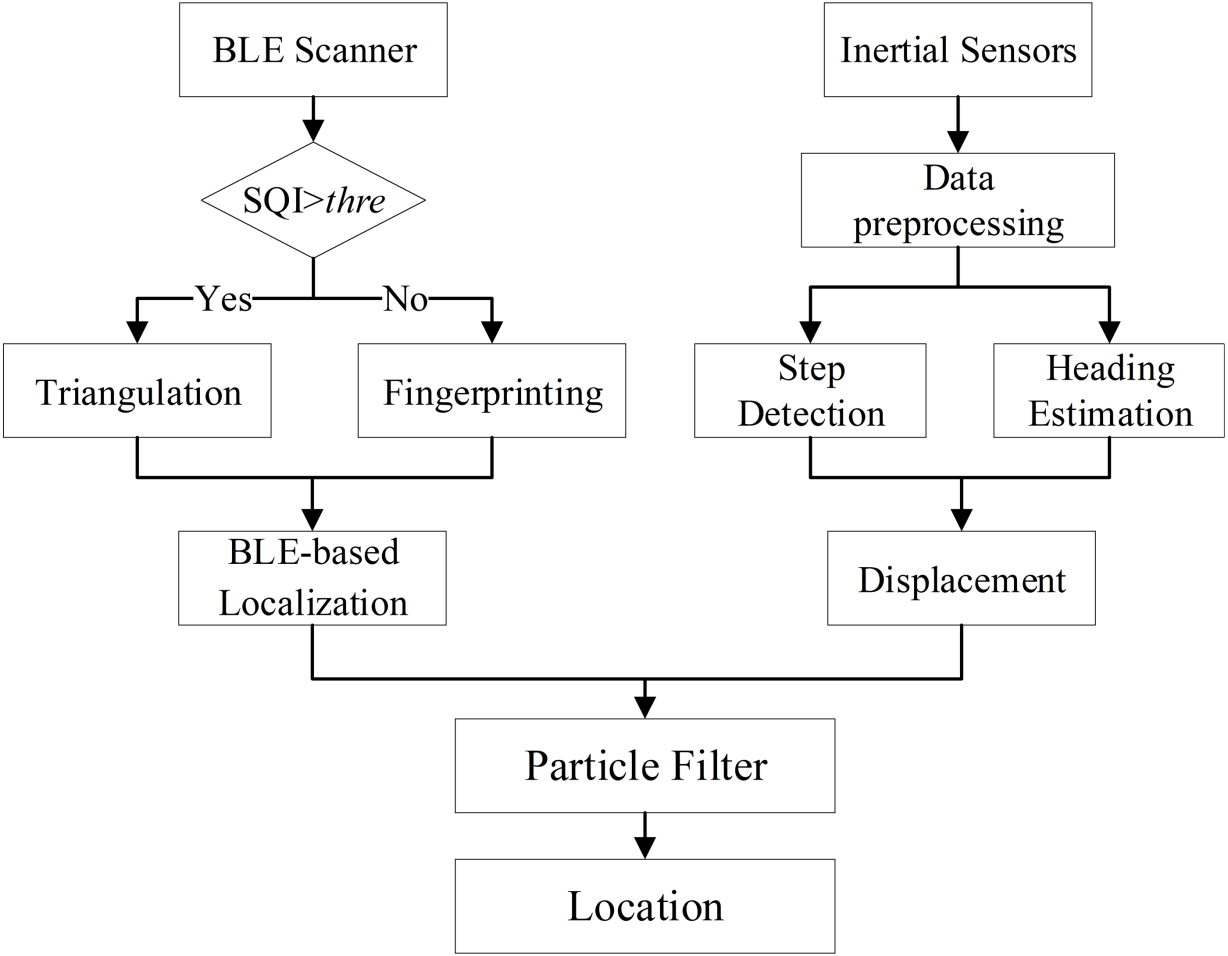
Indoor localization algorithm.

Since we set up a configuration to display the indoor spatial information, the first step in our implementation is tracking the camera pose based on the mobile phone. We propose a novel multi-sensor fusion-based algorithm, which utilizes a particle filter to fuse BLE and PDR. The indoor localization algorithm is presented in Fig. 2.

 The input of the algorithm includes BLE and inertial sensors data. Based on the BLE data, we obtain the BLE-based localization result. Meanwhile, based on the inertial sensors data, we get the displacement estimation result. Then, we use particle filter to fuse these two results. The displacement is used to update the particles for the particle filter.

#### BLE-based indoor localization

BLE is a wireless personal area network technology designed for applications in the healthcare, fitness, beacons, security, and home entertainment industries. Proximity sensing is one of its applications, which also provides a new method for indoor localization. BLE realizes indoor localization by measure the distance between a mobile device and several beacons. The locations of the beacons are known. Specifically, based on the known location of the beacon, the received signal strength (RSS) of the beacon can be used to estimate the distance from the mobile device to the beacon, which can be expressed as Eq. (1):

(1) \begin{eqnarray*}RSS \left( \lambda \right) =RSS \left( {\lambda }_{0} \right) -10\eta \log \nolimits \left( \frac{\lambda }{{\lambda }_{0}} \right) +\mathrm{X}\end{eqnarray*}

where *λ* is the distance from the beacon to device, $RSS \left( \lambda \right) $ is RSS of a beacon, *λ*_0_ is reference distance (normally *λ*_0_ equal to 1m), *η* is the path loss exponent, and X is a zero-mean Gaussian distribution variable with variance ${\sigma }_{\mathrm{X}}^{2}$.

The localization method is called triangulation. If the location of the mobile device is $ \left( x,y,z \right) $, the locations of the beacons are $ \left( {\mathrm{x}}_{\mathrm{i}},{\mathrm{y}}_{\mathrm{i}},{\mathrm{z}}_{\mathrm{i}} \right) $, *i* = 1, 2, …, *N*. *N* is usually more than 3. The measured distances are d_i_. Then, we can get *N* equations: ${\mathrm{d}}_{\mathrm{i}}^{2}=(\mathrm{x}-{\mathrm{x}}_{\mathrm{i}})^{2}+(\mathrm{y}-{\mathrm{y}}_{\mathrm{i}})^{2}+(\mathrm{y}-{\mathrm{y}}_{\mathrm{i}})^{2}$. By solving the *N* equations, we can get the location of the mobile device.

Because of the complexity of indoor environments, the measured distance usually contains error, which makes localization results unreliable. We propose a novel algorithm that improves the localization accuracy of BLE. By the beacon, we can obtain the signal quality index (SQI), which reflects the confidence of estimated distance.

If the SQI is greater than the threshold, we use a triangulation method to calculate the position of the device; otherwise, we use fingerprinting-based method to estimate the location. A weight k-nearest neighbour (WKNN) is adopted for location calculation. The location is calculated by the following equation: *p* = (*w*_*i*_∗*p*_*i*_)(*i* = 1, 2, …, *k*),, where *p* is the estimated location, *w*_*i*_ is the weight, *p*_*i*_ is the location of the ith beacon, *k* is a parameter, which is determined by experiment. *w*_*i*_ is calculated based on the measured distance between the device and beacon:

1) *w*_*i*_ = 1/*d*_*i*_,

2) normalize *w*_*i*_.

#### PDR

**Figure 3:**
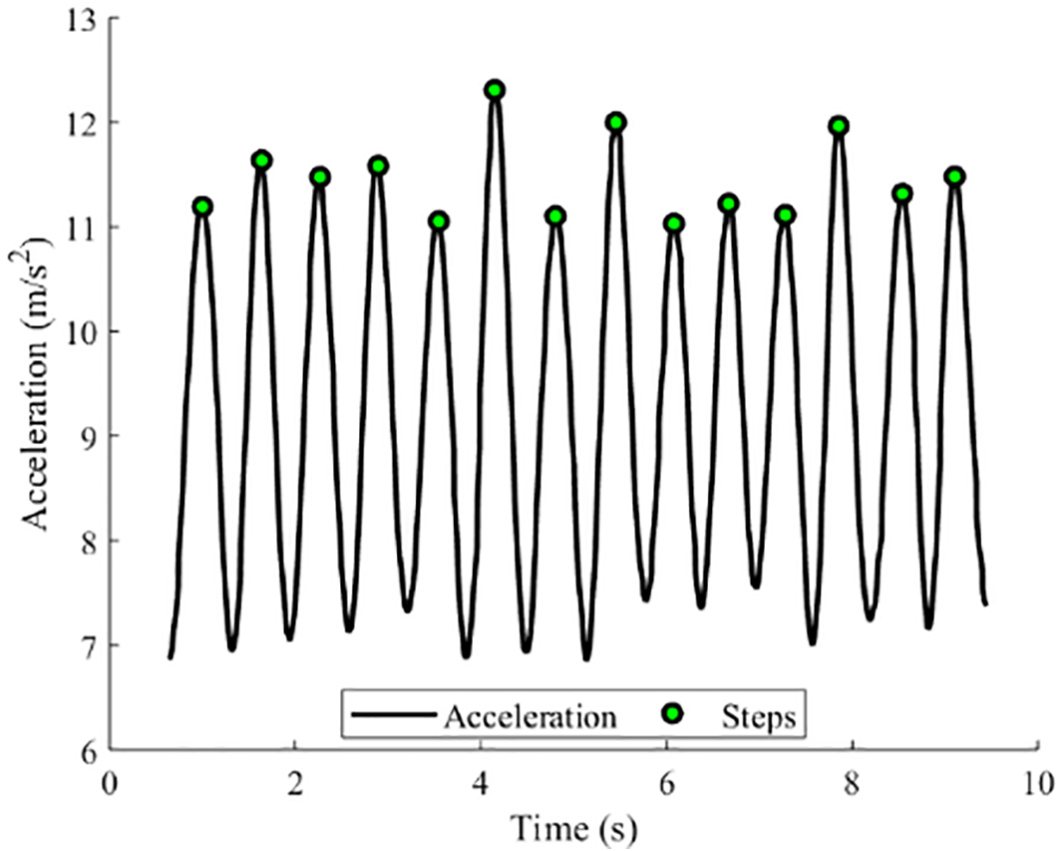
Step detection result.

PDR algorithm is utilized to estimate the displacement. If the previous location is $ \left( x,y \right) $, the next location is calculated as:

(2) \begin{eqnarray*}(x+l\ast n\ast \mathit{cos} \left( h \right) ,~y+l\ast n\ast sin(h))\end{eqnarray*}

where l is the step length, *n* the step number, and the heading. Step number is obtained by the peak detection algorithm (Mladenov & Mock, 2009). The step detection result is shown in Fig. 3. The step length is estimated using the step frequency-based model (Cho et al., 2010): *l* = *a*∗*f* + *b*, where *f* is the step frequency, and (*a*, *b*) are the parameters that can be trained offline.

#### Particle filter-based data fusion

The PF algorithm is based on the sequential Monte Carlo framework, applied to nonlinear and non-Gaussian estimation problems (Arulampalam et al., 2002). A typical particle filter comprises of the following steps:

Initialization: Sampling N particles based on the initial localization result.

Prediction Sampling: Predict a new particle ${\mathrm{p}}^{\mathrm{i}} \left( \mathrm{k}+1 \right) $ for each particle ${p}^{i} \left( k \right) $ based on the prediction function. In this paper, we use PDR to model the user’s movement for the prediction of multiple particles. The new location (${x}_{k+1}^{i},{y}_{k+1}^{i}$) of *i*th particle is updated by

(3) \begin{eqnarray*} \left\{ \begin{array}{@{}c@{}} \displaystyle {x}_{k+1}^{i}={x}_{k}^{i}+\Delta l\cos \nolimits ({h}_{k}+\Delta h)\\ \displaystyle {y}_{k+1}^{i}={y}_{k}^{i}+\Delta l\sin \nolimits ({h}_{k}+\Delta h) \end{array} \right. \end{eqnarray*}

where Δ*l* and Δ*h* are the distance and heading change obtained by PDR, and *h*_*k*_ is the heading at time k.

Importance Sampling: Calculate weights ${\mathrm{w}}^{\mathrm{i}} \left( \mathrm{k}+1 \right) $ for each new particle ${\mathrm{p}}^{\mathrm{i}} \left( \mathrm{k}+1 \right) $*.*

Normalization and Resampling: The weights are normalized and resampled. In the resampling process, particles with low weight are deleted and particles with high weight are duplicated.

In our proposed method, the initial localization is obtained by on BLE-based localization. The particle prediction is realized by PDR algorithm. The weights are calculated based on the distances between the new particles and BLE-based localization results. Based on this online indoor positioning method for AR camera tracking, we fused AR and indoor map for GIS data visualization.

### Augmented reality and indoor map fusion

**Figure 4:**
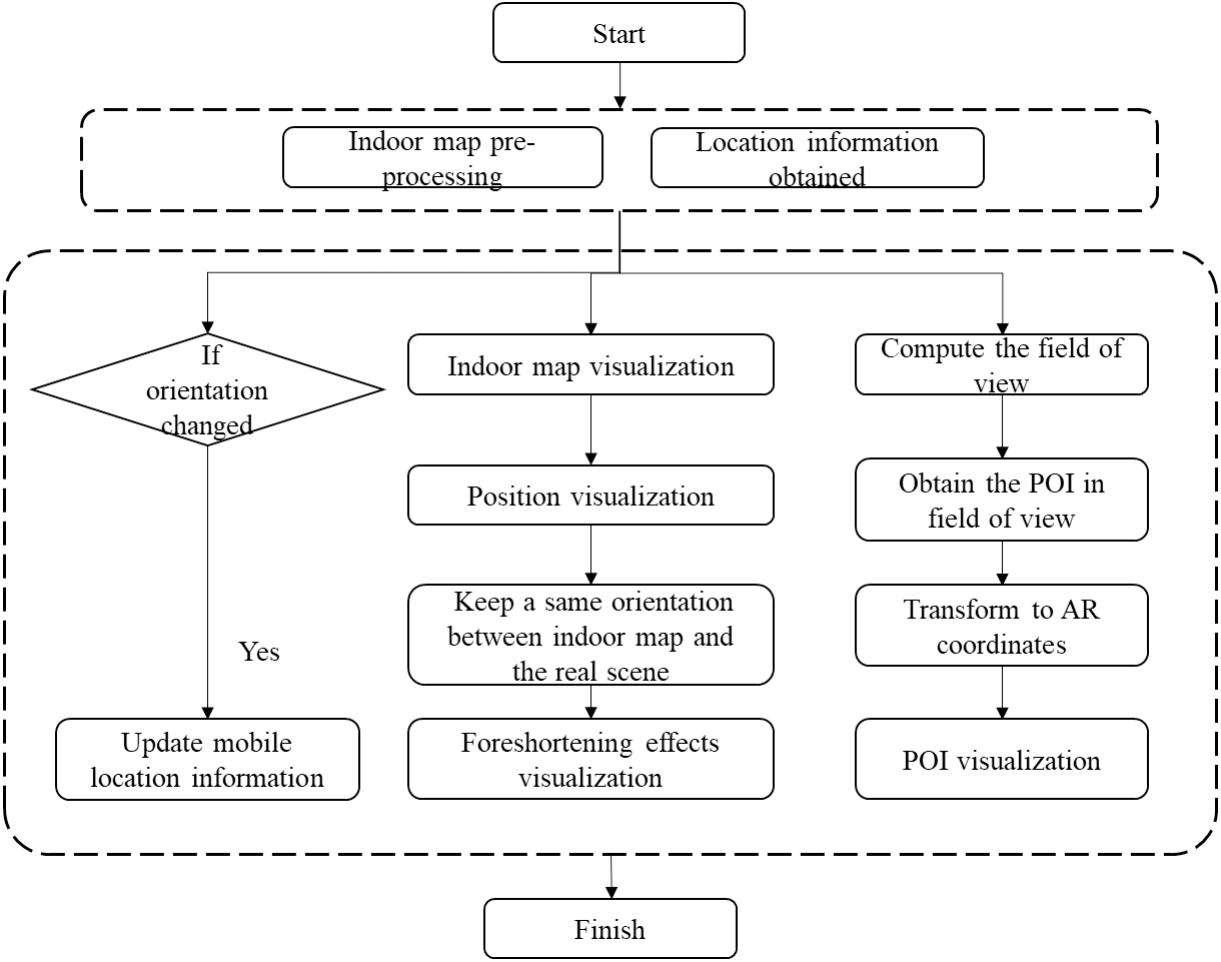
The workflow of AR-GIS visualization fusion.

The workflow of AR-GIS visualization was shown in Fig. 4. We first set up a pre-processing procedure onto indoor map since the GIS information is not adjusted to the visual perspective of the user. During the AR visualization, we extract the extent of the current location from the indoor map and calculate the spatial entities in front of the camera view according to the direction and position of the phone. Then, the spatial entities in front of the camera view are detected and their coordinates transformed from real world to AR view.

#### Indoor map pre-processing

A map constructed of planar features will block the real scene; the indoor map is only composed of line feature that looks transparent in AR view. In order to display attribute information on the indoor map, we keep the text information as a texture and map it onto indoor map. Thus, textual information will align with indoor map whenever an application requires the user to zoom in, zoom out or rotate the map.

The user may be distracted by showing entire map of a large space in an AR view. The displayed part of indoor map should be limited within a certain range of the current location. Thus, we divide indoor map by a regular grid and build an index for map data. Each regular grid cell records the minimum bounding box, transverse step, and longitudinal step. During the AR visualization, we get the current position grid as center, and acquired its surrounding eight grids for displaying. This data structure based on a spatial index of regular grid needs low storage and little communication.

#### AR-GIS visualization

**Figure 5:**
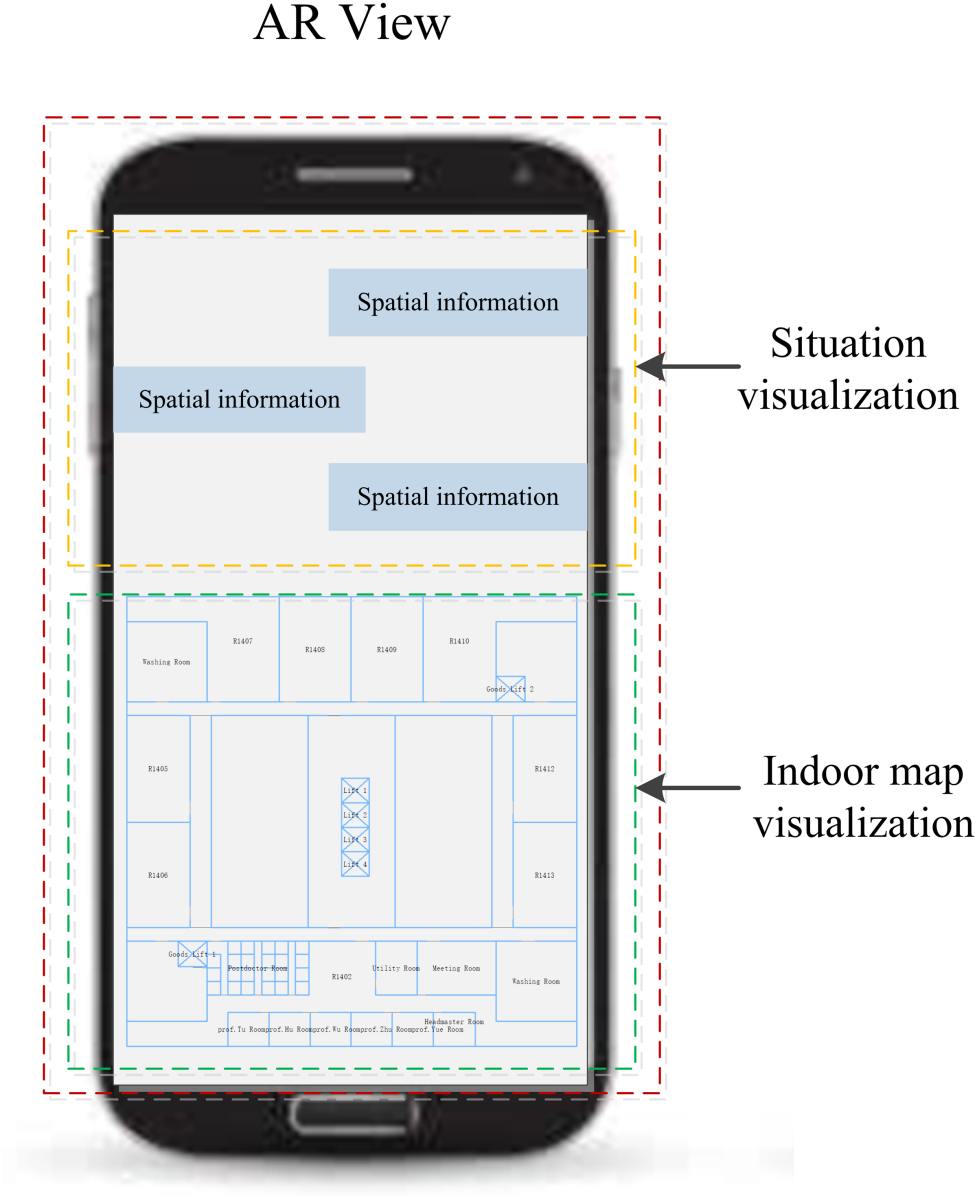
The interface of AR view.

We designed the interface of AR-GIS system that fused the situation and indoor map visualization. As described in Fig. 5, we divided the screen into two parts, which the top one third is displayed the situation information, and the lower two third is rendered the indoor map. Moreover, the indoor positioning result is rendered into the indoor map to show the current user position.

**Figure 6:**
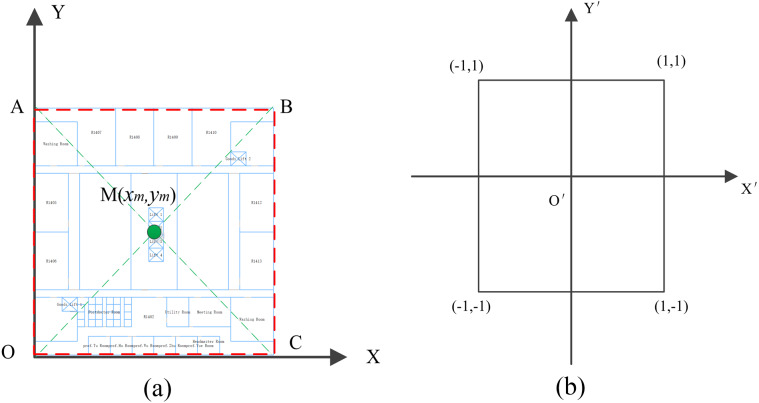
The coordinate transformation. (A) The indoor map coordinate system. (B) The AR view coordinate system.

We transformed all these visual elements into the AR coordinate system. As shown in Fig. 6, Fig. 6A is indoor map coordinate system and Fig. 6B is AR coordinate system.

We acquired the rendered part of indoor map according to the current position, and calculated the bounding box of indoor map ABCO and the center point M(x_m_, y_m_). We put the point *M* as a new original point, and reduce the size of indoor map in accordance with a certain proportion to keep it within the AR view. Considering the indoor map coordinates in Euclidean space [O; x, y], we define the AR coordinate system as [O; x′, y′]. The coordinate transformation can be described as Eq. (4):

(4) \begin{eqnarray*} \left\{ \begin{array}{@{}l@{}} \displaystyle {\mathrm{x}}^{{^{\prime}}}= \frac{(\mathrm{x}-{\mathrm{x}}_{\mathrm{m}})}{\max \nolimits ( \left\vert 2{\mathrm{x}}_{\mathrm{m}} \right\vert , \left\vert 2{\mathrm{y}}_{\mathrm{m}} \right\vert )} \hspace*{10.00002pt}\\ \displaystyle {\mathrm{y}}^{{^{\prime}}}= \frac{(\mathrm{y}-{\mathrm{y}}_{\mathrm{m}})}{\max \nolimits ( \left\vert 2{\mathrm{x}}_{\mathrm{m}} \right\vert , \left\vert 2{\mathrm{y}}_{\mathrm{m}} \right\vert )} \hspace*{10.00002pt}. \end{array} \right. \end{eqnarray*}

Furthermore, we optimized the AR-GIS visualization system from three aspects.

**Figure 7:**
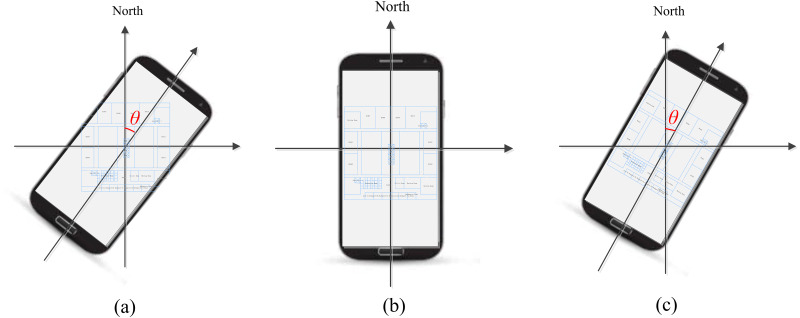
The orientation of indoor map. (A) Mobile phone orientation. (B) The orientation of indoor map changes with mobile phone. (C) The orientation of indoor map changes with the real scene.

The existing map visualization methods do not consider the direction relationship between map and the real world. As shown in Fig. 7B, the orientation of indoor map always changes with mobile phone, not the real scene. Users commonly identify the north by a compass in the map. However, this approach is not suitable for AR visualization. AR explains the spatial context by overlaying digital data onto the users’ view of the real world. The preferable way to make user understand spatial data clearly in an AR view is not by providing more visual elements like a compass, but rendering virtual data that directly matches the real scene. In order to keep a same orientation between indoor map and the real scene, we first obtained the angle between the mobile phone’s orientation and the north. We obtained the orientation and rotation of mobile phone from multiple sensors. Considering clockwise direction as positive direction, when the phone is tilted by an angle *θ* away from the north, we rotate the indoor map by the same angle in the opposite direction. The calculation of angle *θ* in indoor environment needs to account for the deflection angle of the indoor map. As shown in Fig. 7C, the direction of indoor map always matches the real scene whenever the mobile phone rotated, so that users can better understand the map information in AR view.

The foreshortening effects are basic visual rules in the real world. To make the AR visualization more realistic, we rendered the indoor map with foreshortening effects when the phone’s pitch angle changes. We obtain the pitch angle from the sensors in mobile phone, and rotate the indoor map around the X-axis along with the angle change.

**Figure 8:**
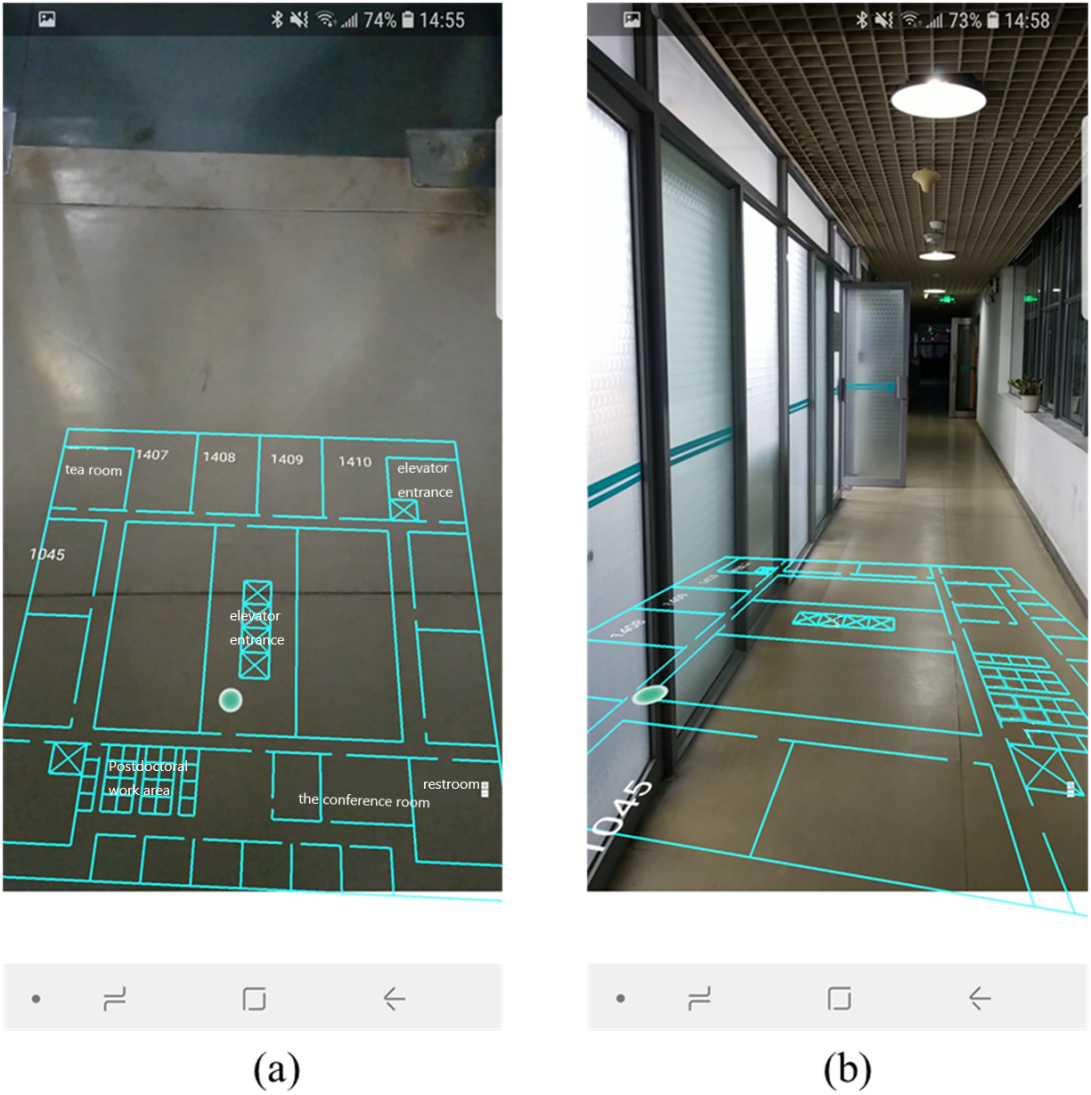
The foreshortening effects of indoor map. (A) The mobile phone is placed horizontally. (B) The mobile phone is placed vertically.

As show in Fig. 8A, when the phone is placed horizontally that the pitch angle is approximately equal to 0°, the user can only see the ground through their phone, so there is no foreshortening effects. The pitch angle is getting bigger along with user lifts the mobile phone, the indoor scene appears in the camera view, and AR map shows the effect of “near big far small” as well (Fig. 8B).

**Figure 9:**
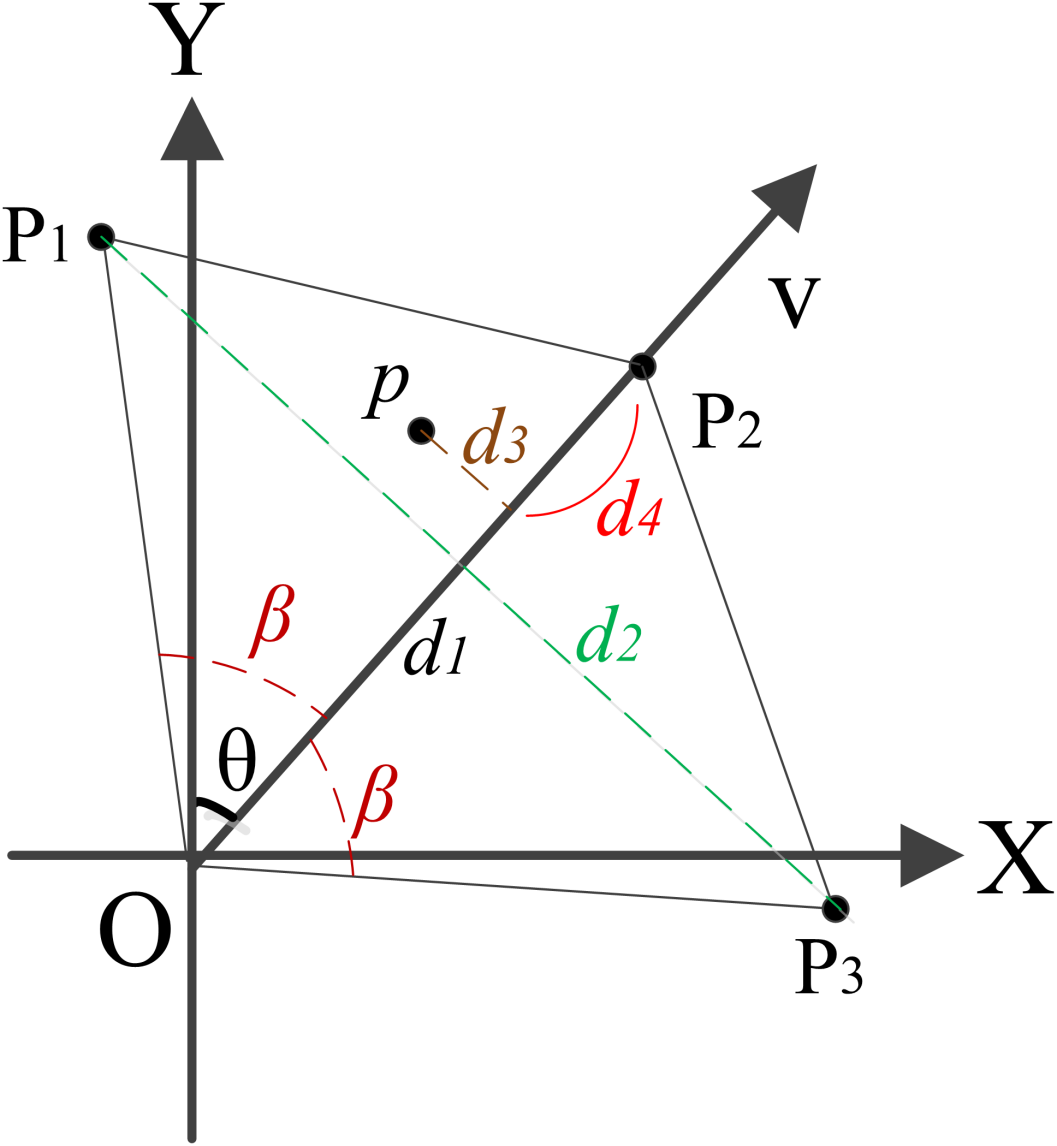
The field of AR view.

We provide the spatial information onto AR view based on the spatial relationship between GIS data and the actual indoor environment. As shown in Fig. 9, O is the current position, V is the camera direction, and θ is the angle to the north. We set two threshold values *d*_1_ and *β*, which identify distance and the angle of vision. Thus, the quadrangle O*p*_1_*p*_2_*p*_3_ is the field of AR view. When an object appears in the quadrangle, its information will be rendered onto AR view. In addition, if the object fall in the triangle Op_1_p_2_, its information will rendered on the left of the AR view. Otherwise, the information will be rendered on the right of the AR view.

We build the virtual and real camera alignment by coordinate transformation. For example, a point *p* in the O*p*_1_*p*_2_, whose screen coordinates of information can be described as:

(5) \begin{eqnarray*} \left\{ \begin{array}{@{}l@{}} \displaystyle x= \frac{ \frac{{d}_{2}}{2} -{d}_{3}}{ \frac{{d}_{2}}{2} } \ast \frac{width}{2} \hspace*{10.00002pt}\\ \displaystyle y= \frac{{d}_{4}}{{d}_{1}} \ast height\hspace*{10.00002pt} \end{array} \right. \end{eqnarray*}

where the width and height are the width and height of screen, *d*_2_ is the distance between *p*_1_ to *p*_3_, *d*_3_ is the perpendicular distance between p to the camera direction v, and the *d*_4_ is the perpendicular distance between *p*_2_ to the perpendicular line segment from *p* and camera direction *v*, where a perpendicular line from *d*_3_ intersects the line from *p* and camera direction *v*.

If the point p in the Op_2_p_3_, the screen coordinates of information can be described as:

(6) \begin{eqnarray*} \left\{ \begin{array}{@{}l@{}} \displaystyle x=width- \frac{ \frac{{d}_{2}}{2} -{d}_{3}}{ \frac{{d}_{2}}{2} } \ast \frac{width}{2} \hspace*{10.00002pt}\\ \displaystyle y= \frac{{d}_{4}}{{d}_{1}} \ast height\hspace*{10.00002pt} \end{array} \right. \end{eqnarray*}

Finally, the indoor map and situational information are rendered on to camera AR view by the coordinate transformation.

## Experimental Results and Analysis

**Figure 10:**
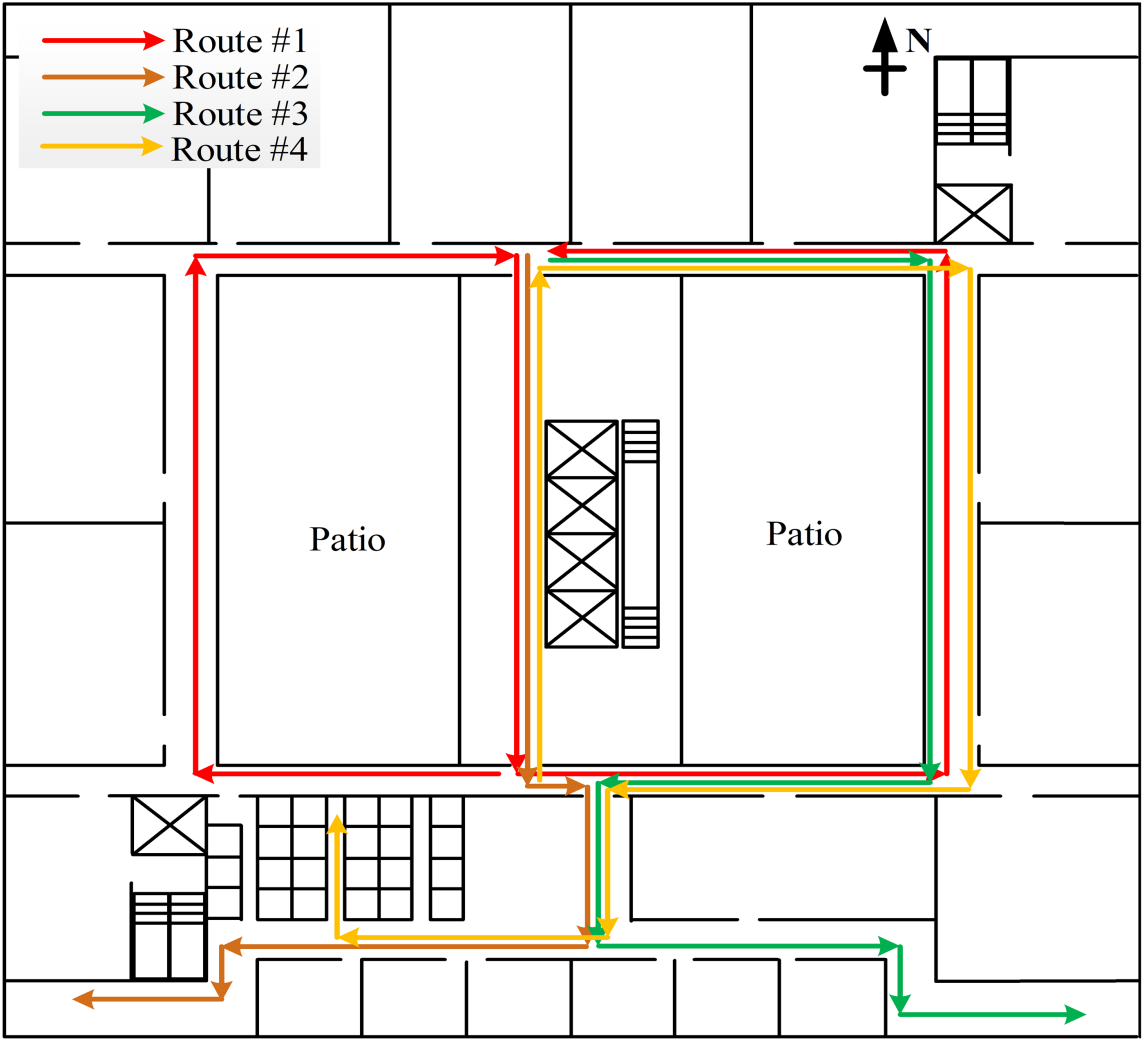
Experiment environment.

Our experiment was operated on an android system mobile phone of Samsung SM-G9500. The AR visualization system was implemented based on OpenES as single-threaded programs. As shown in Fig. 10, the experiment environment is a 52.5 m * 52.5 m floor plan, which includes office area, lift well and public area.

### Indoor positioning

To evaluate the performance of the proposed indoor positioning method, the participants were asked to walk along four routes with the smartphone in the hand. We set some markers with known coordinates along the routes to collect the ground truth data. When participants walk over a marker, they push the button to record the time. The ground truth between the markers is obtained by interpolating the step count. We calculated the positioning error based on the Euclidean distance between the estimated position and the ground truth, and also gave the the Cumulative Distribution Function (CDF) of positioning resulte.

**Figure 11:**
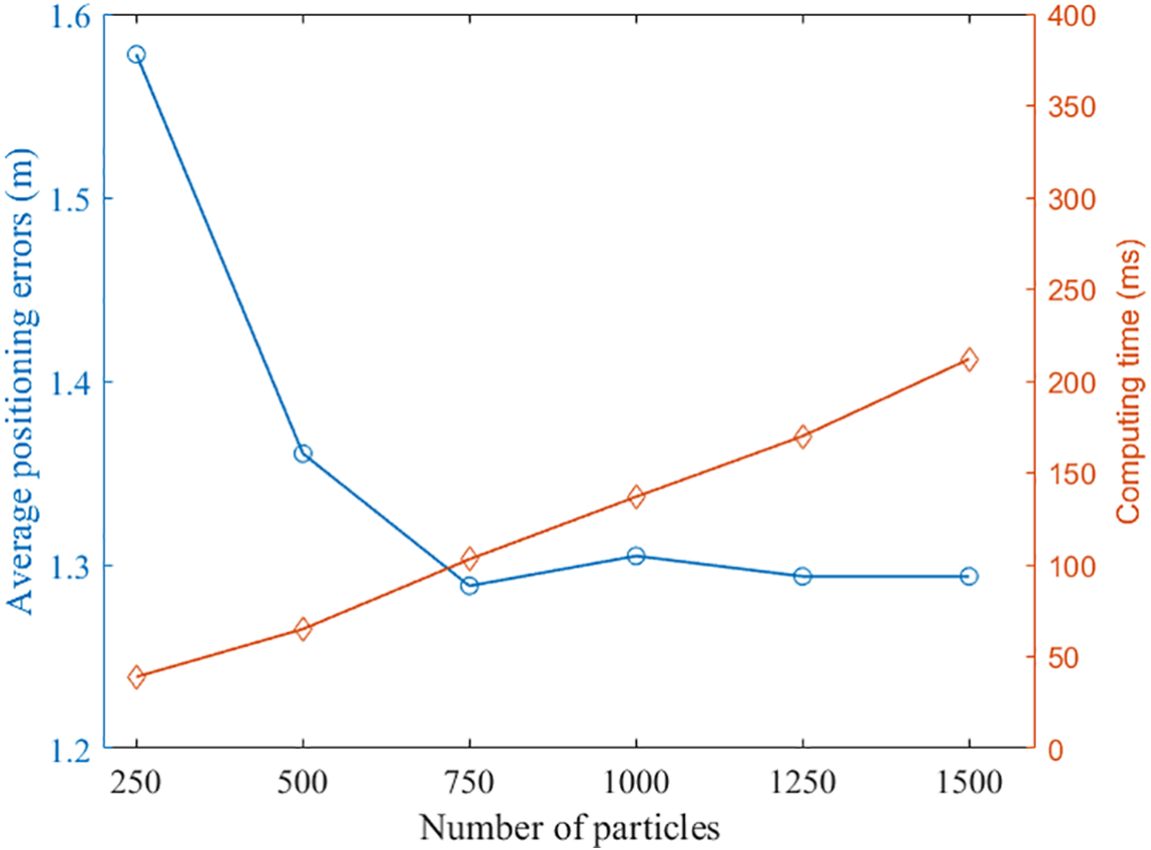
The effect of particles number on the performance of the positioning system.

We evaluated the effect of the number of particles in PF algorithm on the performance of the proposed positioning method. The performance was evaluated in two metrics: average positioning error and computing time. The computing time is a key factor since the proposed positioning system is implemented online on a smartphone. Figure 11 shows the effect of number of the particles on the performance of the positioning system. We can see that with increasing particle number, the average positioning error decreased until the number increased to 750. The computing time increased gradually with the increasing particle number. Therefore, we set the number of particles to 750 in our PF algorithm.

After determine number of particles, we the collected the statistical positioning information of each route. Take route 1 as an example, we captured 143 BLE location, and detected 203 steps. Based on these collected data (143+203), we obtained 210 localization results by using PF algorithm. The statistical positioning information of each route was given in Table 3.

**Figure 12:**
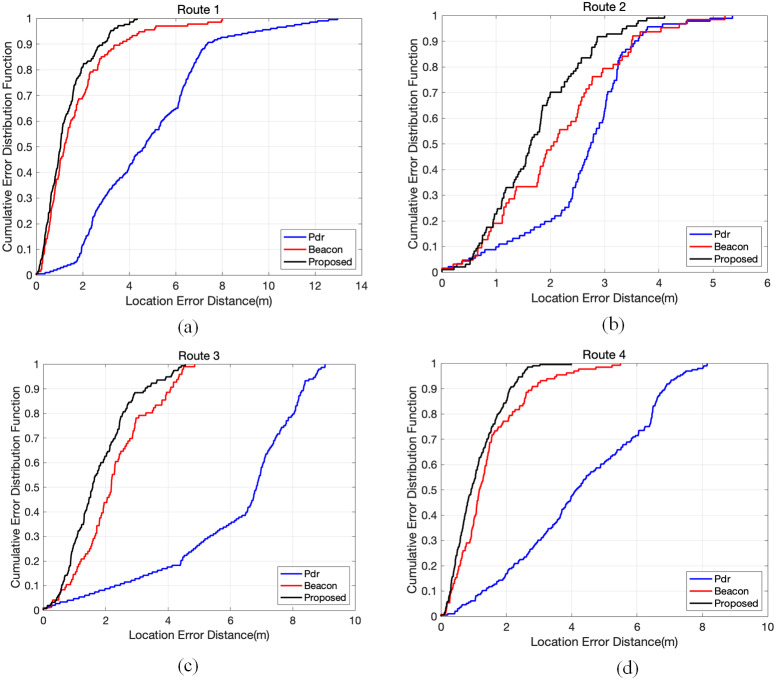
The CDF of different routes. The blue line represents PDR. The red line represents BLE. The black line is the result of proposed method. (A) Route 1, (B) Route 2, (C) Route 3, (D) Route 4.

As shown in Fig. 12, we evaluated the quality of the positioning resulte using the Cumulative Distribution Function (CDF).

Figure 12 shows the CDF of the PDR, Beacon, and PF positioning methods for each route. For route 1, 80% of PF error is within approximately 1.9 m (80% of beacon error is within approximately 2.3 m, and 80% of beacon error is within approximately 6.4 m). For route 2, 80% of PF error is within approximately 2.5 m. For route 3, 80% of PF error is within approximately 2.3 m. For route 4, 80% of PF error is within approximately 1.8 m.

**Table 3:** The statistical positioning information of each route.

	Step number of PDR	Number of BLE location	Number of PF location
Route 1	203	143	210
Route 2	91	63	97
Route 3	148	96	105
Route 4	196	131	204

We also compared the proposed method to PDR and BLE by using average positioning errors. As shown in Table 4, we can see that the average positioning errors of the proposed method for all the routes were less than that of PDR and BLE. For route 1, the average positioning errors of PDR, BLE and proposed were 7.23 m, 1.55 m, and 1.29 m, respectively. The mean errors of the three methods for all the routes were 5.26 m, 1.84 m, and 1.47 m, respectively. In PF-based data fusion, the average positioning error was reduced by 72.04% and 19.89% as compared to PDR and BLE. The positioning results show that the proposed PF-based data fusion algorithm can effectively improve the accuracy of indoor positioning system.

**Figure 13:**
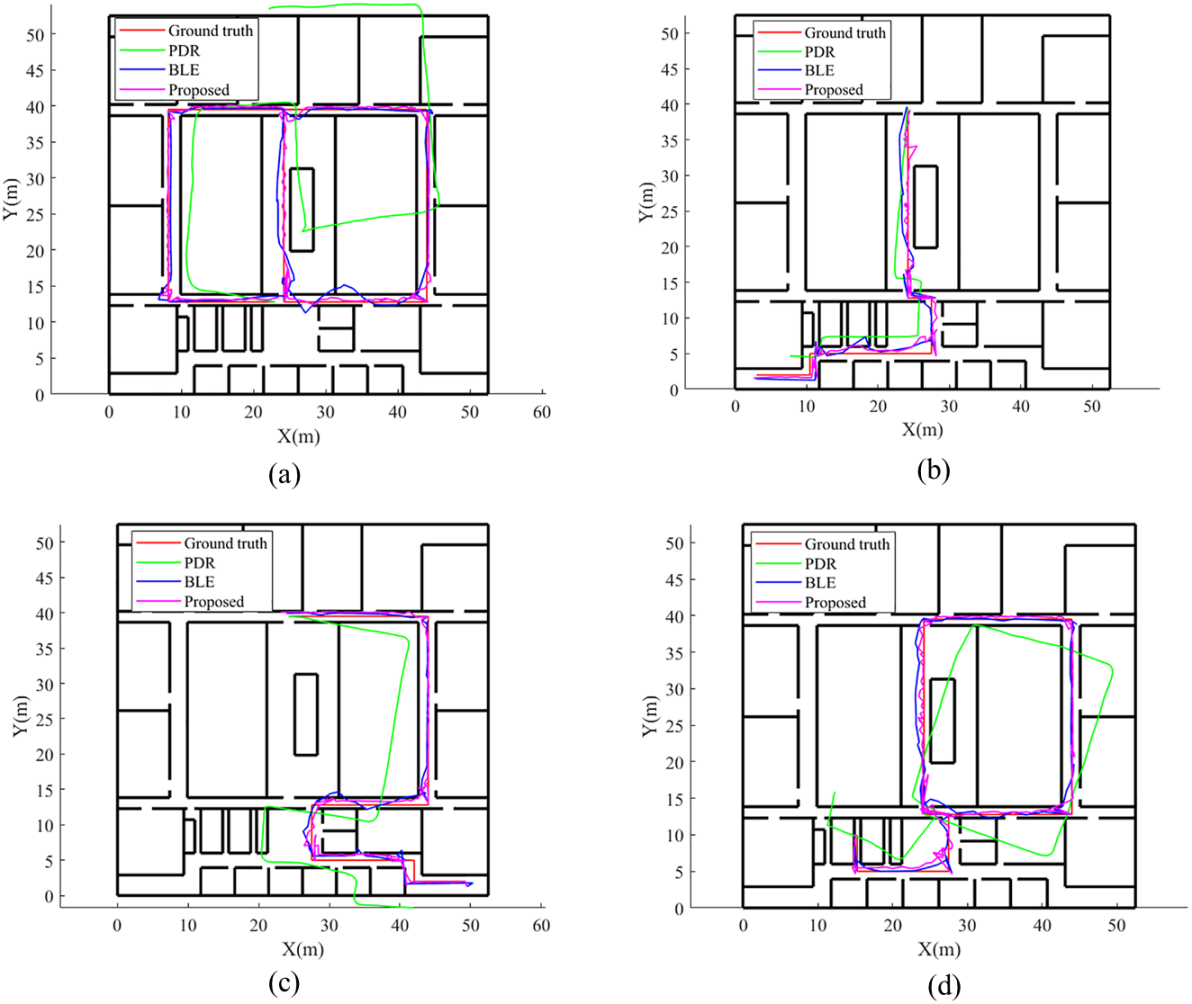
Online positioning result. The red line is ground truth. The green line represents PDR only used. The blue line represents is BLE only used. The purple line is the result of proposed method.

The online positioning results of different positioning methods are shown in Fig. 13 showing Online positioning result. The red line is Ground truth. The green line represents PDR only used. The blue line represents is BLE only used. The purple line is the result of proposed method.

**Table 4:** The average positioning errors (m).

	PDR	BLE	Proposed
Route 1	7.23	1.55	1.29
Route 2	2.6	2.01	1.67
Route 3	6.08	2.08	1.62
Route 4	5.12	1.7	1.3
Mean	5.26	1.84	1.47

We can see that the PDR positioning error is large; the estimated trajectories deviate sharply from the ground truth. The BLE positioning results were more accurate that of PDR due to our proposed algorithm. However, in some areas, BLE has occasional large errors. For example, the bottom right of Route 1 and bottom left of Route 2, show significant deviations. Our proposed PDR and BLE fusion method is more accurate than either PDR or BLE alone. The estimated trajectories are consistent with the ground truth.

### AR-GIS visualization

We walked with the smartphone to the test the AR-GIS visualization, and recorded the spatial information visualization results. The video of mobile augmented reality system was uploaded on the website: https://youtu.be/XNhpaIWk1IQ.

**Figure 14:**
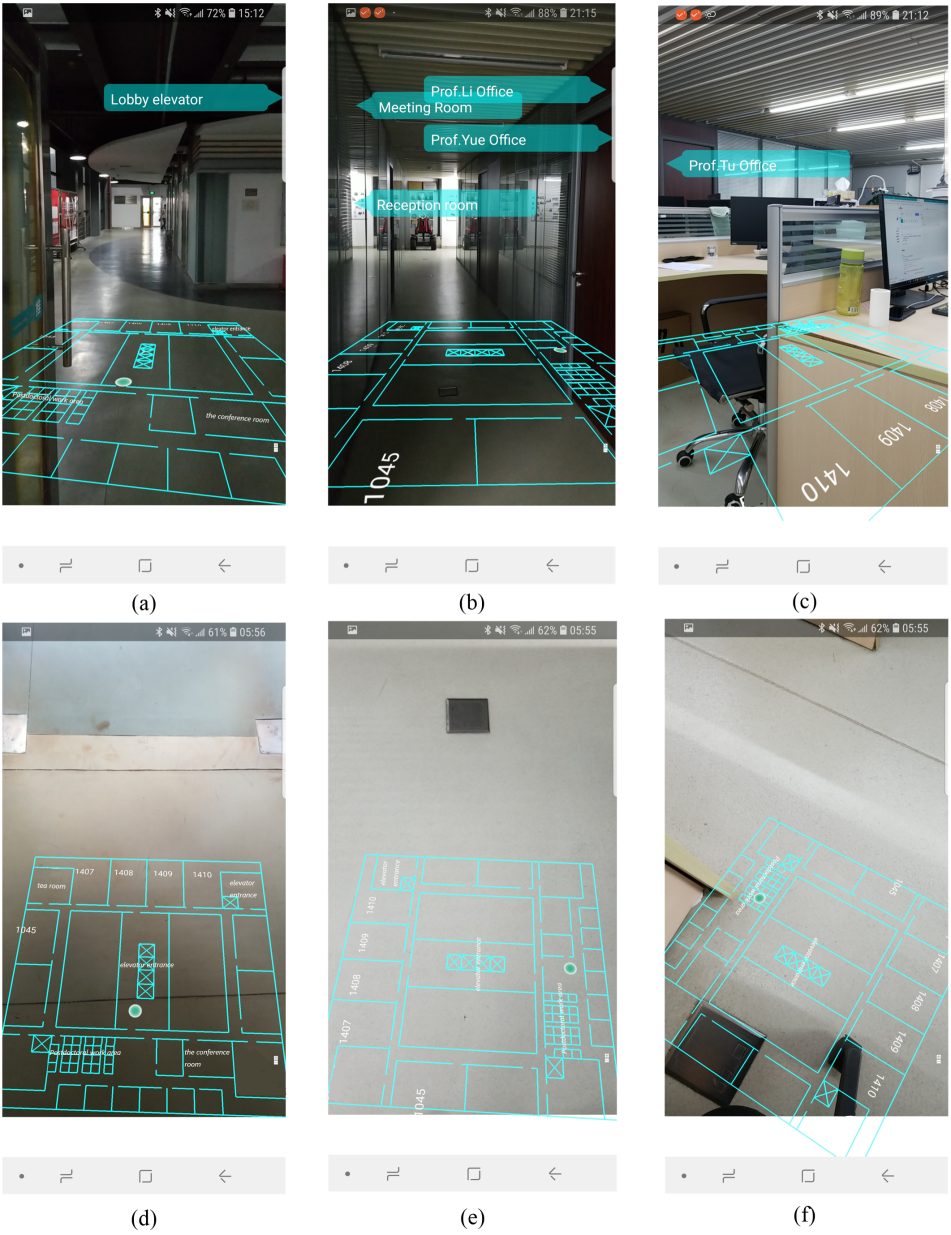
The results of AR-GIS visualization.

The AR visualization effects are illustrated in Fig. 14, where each column represents a different position. The first row presents the AR visualization effect when the mobile phone is upright. It consists of situational information, the indoor map and localization results. In Fig. 14A, the lobby elevator is on the left side of the current position, so the text was rendered on the left of AR view. In Fig. 14B three targets were detected, the texts were displayed from top to bottom according to the distance from far to near. In Fig. 14C, the office of Prof.Tu is on the right of the current position, so the label was rendered on the right of the AR view.

The second row (Figs. 14D, 14E, 14F) shows the AR visualization results when the mobile phone is placed horizontally. User can clearly perceive the indoor space and current position from this perspective.

We discuss our approach as compared with two state-of-the-art AR systems: (Xu et al., 2020; Khan et al., 2019). Xu et al. (2020) proposed an AR system for indoor/outdoor navigation. The AR positioning signals are corrected continuously by the ground-truth 3D indoor/outdoor walkability network in a 3D model. However, it is necessity for their tool to implement approaches through is a manual 3D drawing of indoor walkable space. Khan et al. (2019) presented an AR system based on the correspondences between images. However, failure becomes more likely due to frequent changes in indoor environment and because reliable reference points are unavailable. Moreover, the AR application (Xu et al., 2020) only offer the position spot onto the AR view, and the AR system (Khan et al., 2019) only labels the facility’s name along the pathway. These two methods can only provide local spatial in-formation in fixed location for users. Compare with them, our AR and indoor map fusion technique links rich indoor spatial information to real world scenes. Figure 14 presents a high-quality AR-GIS system that integrates rich spatial information with the real world tightly and rationally. It is not only technical but also aesthetical for conveying spatial information.

## Conclusions and Future Work

In this paper, we designed a method for fusing AR view and indoor map in changing environments during AR-GIS visualization that also considers the changes of the AR view when the pose of the mobile phone shifts. We presented an innovative indoor positioning approach that fuses BLE and PDR to enhance the accuracy of AR camera tracking. The experiments in an office building of Shenzhen University demonstrate that dynamic AR-GIS visualization techniques can display rich spatial information in real time on mobile phones, while preserving a high accuracy in the AR-GIS fusion. The AR content includes current position, indoor map and spatial information, which change as the real scene changes. The layout of spatial information is rendered reasonable in AR view. This AR-GIS visualization will be clear and easy to understand, which effectively optimizes and enriches the spatial information in the visualization of indoor environment.

Though the AR-GIS system is shown indoor map onto the AR view, it can’t adapt to the change of the scene size. Thus, in future work, we will try to process indoor map data and improve the visualization technique method to realize the adaptive AR visualization. Besides, the current application only labels the name of indoor scene, which can be further advanced with more details about the spatial information. Furthermore, the smoothing algorithm can be applied to enhance the accuracy of the device’s position.

## Supplemental Information

10.7717/peerj-cs.704/supp-1 Supplemental Information 1 AR-GIS system demo

10.7717/peerj-cs.704/supp-2 Supplemental Information 2 The indoor positioning code and codebook This is an online indoor positioning method that fuses the Bluetooth low energy (BLE) and pedestrian dead-reckoning (PDR) for AR camera tracking. The read_json.m is reading the pdr and BLE data for positoning and build Route 1. The read_json2.m is reading the pdr and BLE data for positoning and build Route 2. The read_json3.m is reading the pdr and BLE data for positoning and build Route 3. The read_json4.m is reading the pdr and BLE data for positoning and build Route 4. The plotline1.m is use for drawing the CDF figure.

10.7717/peerj-cs.704/supp-3 Supplemental Information 3 The raw data for indoor positioning

## Additional Information and Declarations

### Data Availability

The following information was supplied regarding data availability: The indoor positioning code is available in the Supplemental Files.
